# A Novel Mechanism of Tumour Communication: Malignant Extracellular Vesicles‐Induced Aggressiveness in Benign Cells and Melatonin‐Driven Reversal

**DOI:** 10.1111/jcmm.71309

**Published:** 2026-08-11

**Authors:** Barbara Maria Frigieri, Guilherme Silva Bruno Barbosa, Adriana Alonso Novais, Caroline Patini de Rezende, Fausto Almeida, Vinicius Augusto Simão, Mari Cleide Sogayar, Luiz Gustavo de Almeida Chuffa, Russel J. Reiter, Debora Aparecida Pires de Campos Zuccari

**Affiliations:** ^1^ Cancer Molecular Research Laboratory (CMRL), Faculdade de Medicina de São José do Rio Preto—FAMERP São José do Rio Preto Brazil; ^2^ Institute of Biosciences, Letters and Exact Sciences IBILCE/ UNESP São José do Rio Preto Brazil; ^3^ Department of Immunology and Biochemistry Ribeirão Preto Medical School—USP, Cidade Universitária Ribeirão Preto Brazil; ^4^ Department of Structural and Functional Biology, Institute of Biosciences São Paulo State University (UNESP) Botucatu Brazil; ^5^ Nucel Group for Cellular and Molecular Therapy São Paulo Medical School—USP São Paulo Brazil; ^6^ Biochemistry Department, Chemistry Institute University of São Paulo São Paulo Brazil; ^7^ Department of Cell Systems and Anatomy Joe R and Teresa Lozano Long School of Medicine, UT Health San Antonio San Antonio Texas USA

**Keywords:** breast cancer, extracellular vesicles, melatonin, phenotypic remodelling, tumour microenvironment

## Abstract

Extracellular vesicles (EVs/EV) are key mediators of intercellular communication and influence proliferation, migration, and metabolic reprogramming. In breast cancer, EVs released by malignant cells carry bioactive molecules capable of altering the behaviour of surrounding cells. However, it remains uncertain whether these vesicles can directly induce a more aggressive phenotype in benign mammary cells. Melatonin, known for its oncostatic properties, regulates proliferation, metabolism, and signalling pathways associated with tumour progression and may modulate EV‐mediated intercellular communication. This study evaluated whether EVs derived from four breast cancer cell lines (MCF‐7, MDA‐MB‐231, MDA‐MB‐453 and HCC70), treated or not with melatonin, can modify the phenotype of benign MCF10A cells. EVs were isolated from conditioned media and co‐cultured with benign cells. Assays of proliferation, viability, colony formation, migration, lactate production and immunofluorescence were performed to assess EV‐mediated effects. EVs derived from malignant cells promoted a more aggressive phenotype in benign cells, as evidenced by increased proliferation, migration, and phenotypic remodelling. In contrast, EVs derived from melatonin‐treated malignant cells were associated with reduced lactate production and attenuation of pro‐tumoural features. Overall, these findings demonstrate that tumour‐derived EVs can induce aggressive traits in benign mammary cells and suggest that melatonin exposure in donor cells modulates EV‐mediated effects, attenuating their pro‐tumoural influence.

## 1 Introduction

Breast cancer remains the most prevalent malignant neoplasm worldwide and represents a major public health challenge due to its high morbidity, mortality, and therapeutic resistance, despite considerable advances in diagnosis and treatment [1, 2, 3]. The classification of breast tumours according to histological grade and molecular subtypes—Luminal A, Luminal B, HER2‐overexpressing and triple‐negative breast cancer (TNBC)—has supported the development of personalized therapeutic strategies. However, this framework does not fully capture the complex and dynamic interactions within the tumour microenvironment [4, 5].

Extracellular vesicles (EVs), nanoscale membrane‐bound structures released by cells, are key mediators of intercellular communication in both physiological and pathological contexts. By transporting proteins, lipids, nucleic acids, and metabolites, EVs regulate critical processes involved in tumour progression, including proliferation, invasion, stromal remodelling, and metastatic dissemination. In breast cancer, EVs act not only as biomarkers but also as active effectors capable of reprogramming recipient cells. However, it remains unclear whether EVs derived from malignant breast cancer cells can directly induce aggressive phenotypic traits in benign mammary cells, representing an important gap in the current understanding of tumour evolution [6, 7, 8].

Melatonin, a hormone primarily synthesized by the pineal gland and also produced in mitochondria and peripheral tissues, has been extensively studied for its pleiotropic regulatory functions. It exerts well‐documented antitumoural effects, including antioxidant, anti‐inflammatory, antiproliferative, antimetastatic, and pro‐apoptotic actions. Increasing evidence indicates that melatonin can modulate key signalling pathways and metabolic processes involved in tumour progression. However, whether melatonin can influence EV‐mediated intercellular communication, particularly through modulation of EV cargo and function, remains poorly understood [9, 10, 11, 12, 13, 14, 15, 16].

To address this gap, the present study investigated whether EVs derived from malignant breast cancer cell lines can alter the phenotype of benign mammary cells and whether exposure of malignant cells to melatonin modifies the effects mediated by these EVs. By exploring the interaction between tumour‐derived EVs and melatonin, this study contributes to the understanding of EV‐mediated communication in cancer and highlights the potential of targeting EV‐associated signalling pathways as a therapeutic strategy.

## 2 Materials and Methods

### 2.1 Treatments

Melatonin (Sigma‐Aldrich, St. Louis, MO, USA) was used at a concentration of 1 mM, based on previously standardized conditions [17]. The compound was diluted in a solution containing 50% phosphate‐buffered saline (PBS) and 50% absolute ethanol and applied to the cells for 24 h.

### 2.2 Experimental Groups

Breast cancer cell lines MCF‐7, HCC70, MDA‐MB‐231 and MDA‐MB‐453 were used in this study. MCF‐7 and HCC70 cells were obtained from the laboratory's cell bank, while MDA‐MB‐231 and MDA‐MB‐453 cells were provided by the Brazilian Biosciences National Laboratory (LNBio). The MCF10A cell line was provided by Prof. Dr. Thomas Ong (University of São Paulo, USP) and cultured at the NUCEL Laboratory (USP).

MCF‐7, HCC70 and MDA‐MB‐231 cells were cultured in Dulbecco's Modified Eagle Medium (DMEM [high glucose, with phenol red, Gibco]) supplemented with 10% foetal bovine serum (FBS) and 1% penicillin–streptomycin–amphotericin solution (Sigma–Aldrich). MDA‐MB‐453 cells were cultured in RPMI 1640 medium containing phenol red, supplemented with 10% FBS, 1% penicillin–streptomycin–amphotericin, and 2 mM L‐glutamine (Thermo Fisher Scientific). All breast cancer cell lines were maintained at 37°C in a humidified atmosphere with 5% CO_2_.

For the experimental design, each breast cancer cell line was divided into two conditions: (i) untreated cells (control) and (ii) cells treated with melatonin for 24 h. Extracellular vesicles (EVs) were subsequently isolated from the conditioned media of both conditions to evaluate the effects of melatonin exposure on EV‐mediated signalling.

### 2.3 Isolation and Characterization of Extracellular Vesicles

Extracellular vesicles (EVs) were isolated from the conditioned media of breast cancer cell lines cultured under untreated or melatonin‐treated conditions. Cells were grown to approximately 80% confluence and, when applicable, treated with 1‐mM melatonin for 24 h in culture medium supplemented with EV‐depleted foetal bovine serum.

EVs were isolated by differential ultracentrifugation. Briefly, cell culture supernatants were first subjected to centrifugation at 2000× *g* for 15 min at 4°C to remove cells and debris. The supernatants were then centrifuged at 15,000× *g* for 15 min at 4°C (Thermo Fisher Sorvall Legend RT centrifuge) to eliminate larger vesicles and particles.

Subsequently, the samples were filtered through a 0.22‐μm filter and ultracentrifuged at 100,000× *g* for 1 h at 4°C using an Optima TLX ultracentrifuge (Beckman Coulter). The resulting EV pellets were resuspended in PBS and stored for downstream analyses.

EV characterization was performed using nanoparticle tracking analysis (NTA) to determine size distribution and particle concentration.

### 2.4 Nanoparticle Tracking Analysis (NTA)

EV size distribution and concentration were determined using nanoparticle tracking analysis (NTA) with a NanoSight NS300 system (Malvern Panalytical, UK) equipped with NTA software version 3.1 (Build 3.1.45).

Samples were diluted 1:500 in calcium‐ and magnesium‐free phosphate‐buffered saline (PBS) and analysed at 37°C using a sCMOS camera. For each sample, five 30‐s videos were recorded and subsequently analysed to determine particle size distribution and concentration based on Brownian motion and light scattering properties.

### 2.5 Transmission Electron Microscopy (TEM)

Transmission electron microscopy (TEM) was performed to assess EV morphology, following previously described protocols in accordance with MISEV guidelines [18].

Briefly, EV samples were diluted in PBS (100 μL), and a 20‐μL aliquot was placed onto carbon‐coated copper grids and incubated for 1 min at room temperature. Excess liquid was removed using filter paper. The samples were then negatively stained with 2% phosphotungstic acid (PTA) for 5 min at room temperature. Subsequently, the grids were fixed with 2% glutaraldehyde for 5 min and washed three times with PBS.

Images were acquired using a Tecnai Spirit transmission electron microscope (FEI Company) operated at 80 kV, at a magnification of 140,000×.

### 2.6 Cell Culture of MCF10A

MCF10A cells, derived from benign mammary epithelium, were cultured in DMEM/F12 medium supplemented with 10% horse serum, 20 ng/mL epidermal growth factor (EGF), 0.5 μg/mL hydrocortisone, 10 μg/mL insulin, and 1% penicillin–streptomycin–amphotericin solution.

Cells were maintained at 37°C in a humidified atmosphere containing 5% CO_2_.

For experimental treatments, MCF10A cells were exposed to extracellular vesicles (EVs) derived from breast cancer cell lines under untreated or melatonin‐treated conditions, as described in Table 1.

**TABLE 1:** Experimental conditions and corresponding extracellular vesicle (EV) sources.

Group	Treatment
MCF‐7 (Ctrl) EVs	Non‐treated MCF‐7 cells
MCF‐7 (Mel) EVs	Treated MCF‐7 cells
MDA‐MB‐231 (Ctrl) EVs	Non‐treated MDA‐MB‐231 cells
MDA‐MB‐231 (Mel) EVs	Treated MDA‐MB‐231 cells
MDA‐MB‐453 (Ctrl) EVs	Non‐treated MDA‐MB‐453 cells
MDA‐MB‐453 (Mel) EVs	Treated MDA‐MB‐453 cells
HCC70 (Ctrl) EVs	Non‐treated HCC70 cells
HCC70 (Mel) EVs	Treated HCC70 cells

Abbreviations: Ctrl, non‐treated cells; Mel, melatonin‐treated cells.

### 2.7 EVs in MCF10A Cell Culture

EVs were quantified and standardized to a concentration of 1 × 10^7^ particles per condition prior to their addition to MCF10A cell cultures, ensuring that all functional assays were performed using equivalent numbers of vesicles across experimental groups. However, EV uptake by recipient cells was not directly quantified, and therefore differences in internalization efficiency between conditions cannot be excluded.

Although uptake was not directly quantified, EVs were standardized by particle number and applied under identical experimental conditions, which reduces—but does not completely eliminate—the possibility of differences in internalization efficiency between groups.

### 2.8 Viability Assay (MTT)

Cell viability was assessed using the MTT assay [3‐(4,5‐dimethylthiazol‐2‐yl)‐2,5‐diphenyltetrazolium bromide]. MCF10A cells were seeded in 96‐well plates at a density of 5 × 10^3^ cells per well in complete culture medium.

After 24 h, the medium was replaced with serum‐free medium and cells were incubated for an additional 24 h. Subsequently, the medium was replaced with complete medium containing 10% foetal bovine serum, and cells were treated with extracellular vesicles (EVs) derived from untreated or melatonin‐treated cells for 24 h.

Following treatment, MTT reagent was added and cells were incubated for 4 h at 37°C in a humidified atmosphere with 5% CO_2_ to allow for formazan crystal formation. The medium was then removed, and the crystals were solubilized in dimethyl sulfoxide (DMSO). Absorbance was measured at 570 nm using a microplate reader.

All experiments were performed in triplicate. Cell viability was calculated as a percentage relative to control using the equation: viability (%) = (A/B) × 100, where A represents the absorbance of treated samples and B represents the absorbance of control samples.

### 2.9 Cell Migration Assay

Cell migration was evaluated using a transwell assay, as previously described by [19], MCF10A cells were initially seeded in 24‐well plates at a density of 5 × 10^4^ cells per well in 500 μL of complete medium and allowed to adhere for 24 h. Subsequently, cells were treated with extracellular vesicles (EVs) derived from untreated or melatonin‐treated cells for an additional 24 h.

After treatment, cells were trypsinized and 3 × 10^4^ cells per condition were resuspended in 200 μL of serum‐free medium and seeded into transwell inserts with 8‐μm pore polycarbonate membranes (BD Biosciences, San Jose, CA, USA). Inserts were placed into 24‐well plates containing 500 μL of complete medium supplemented with 10% serum in the lower chamber.

Cells were incubated at 37°C in a humidified atmosphere with 5% CO_2_ to allow migration. After incubation, non‐migrated cells were removed from the upper surface of the membrane, and migrated cells on the lower surface were fixed with 4% paraformaldehyde for 30 min. Cells were then stained with crystal violet for 60 min, washed with PBS to remove excess dye, and air‐dried.

Migrated cells were visualized under a microscope, and images were captured from five random fields per insert. The number of migrated cells was quantified for each condition.

### 2.10 Colony Formation Assay

Cells were seeded in 6‐well plates at a density of 16 × 10^2^ cells/well in 1 mL of complete medium. After 24 h, the medium was replaced and EVs were added. Every 2 days, the medium and treatments were changed. After 14 days, colonies were fixed with methanol for 15 min and stained with 0.1% crystal violet for 10 min. Finally, the plates were washed, and colonies were photographed and counted (Figure 3III).

### 2.11 Lactate Formation

The cells were placed in 24‐well plates at a concentration of 5 × 10^4^ cells/well in 500 μL, in triplicate. After 24 h, the medium was replaced, and treatments were added. Following another 24 h, the supernatant was removed and stored at −80°C. After thawing, lactate levels were measured using Labtest enzyme kits (Labtest Diagnóstica S.A., Lagoa Santa—MG/BR) according to the manufacturer's instructions. Absorbance was read on the spectrophotometer, and lactate concentrate was calculated as: Lactate (mg/dL) = (Absorbance of sample/Absorbance of standard) × 40 (Figure 3IV).

### 2.12 Immunofluorescence Analysis

MCF10A cells were seeded in 24‐well plates containing sterile glass coverslips at a density of 5 × 10^4^ cells per well in 1 mL of culture medium and allowed to adhere.

Cells were then treated with extracellular vesicles (EVs) derived from untreated or melatonin‐treated cells. Following treatment, cells were fixed with 4% paraformaldehyde and subjected to immunofluorescence staining for Ki‐67 (1:100, Abcam) and cleaved caspase‐3 (1:100, Abcam). Alexa Fluor 488‐conjugated anti‐rabbit secondary antibody (Invitrogen) was used for detection.

Nuclei were counterstained with DAPI (1:10,000). Fluorescence images were acquired using an EVOS M5000 fluorescence microscope.

### 2.13 Statistical Analysis

All experiments were performed in duplicate or triplicate, and data are presented as mean ± standard error of the mean (SEM). Statistical comparisons among multiple groups were performed using one‐way analysis of variance (ANOVA), followed by Tukey's post hoc test.

A *p* < 0.05 was considered statistically significant. All analyses were conducted using GraphPad Prism version 9.0 (GraphPad Software).

## 3 Results

### 3.1 Isolation, Identification and Characterization of EVs From Breast Cancer Cell Lines Under Control and Melatonin Treatment

Extracellular vesicles (EVs) were isolated from the conditioned media of untreated and melatonin‐treated breast cancer cell lines by ultracentrifugation, as described in Section 2.3.

**FIGURE 1:**
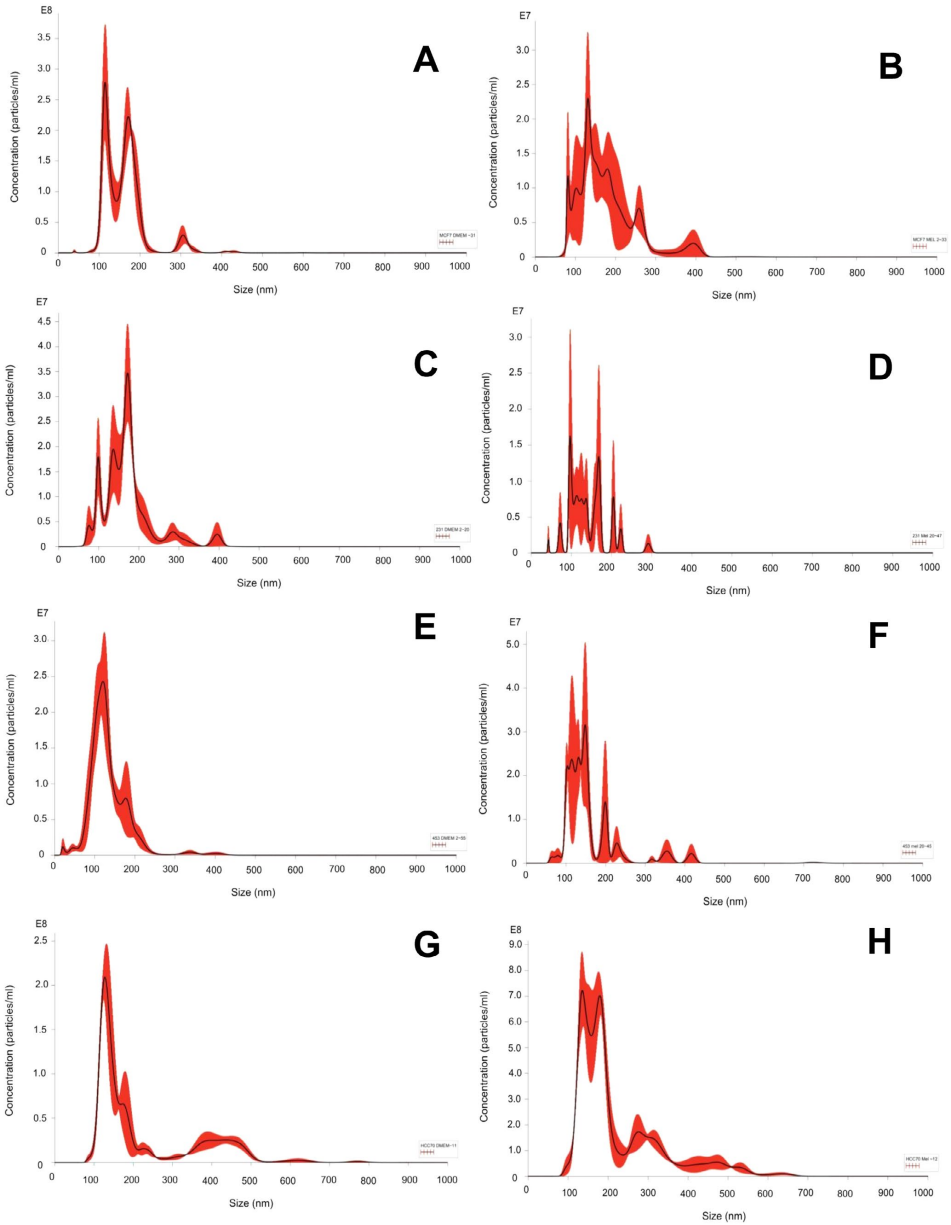
Size distribution of extracellular vesicles (EVs) derived from breast cancer cell lines, measured by nanoparticle tracking analysis (NanoSight NS300, Malvern Panalytical, UK). EVs were isolated from the conditioned medium of untreated or melatonin‐treated cells: (A) MCF‐7 (untreated), (B) MCF‐7 (melatonin‐treated); (C) MDA‐MB‐231 (untreated), (D) MDA‐MB‐231 (melatonin‐treated); (E) MDA‐MB‐453 (untreated), (F) MDA‐MB‐453 (melatonin‐treated); (G) HCC70 (untreated), (H) HCC70 (melatonin‐treated). EV size ranged from 30 to 160 nm. Data are presented as mean ± SEM (*n* = 3 independent experiments).

EV characterization was performed using nanoparticle tracking analysis (NTA) and transmission electron microscopy (TEM). NTA measurements indicated that EV sizes ranged from 117.0 to 154.1 nm (Figure 1 and Table 2), consistent with ISEV guidelines. No significant differences in size distribution were observed between EVs derived from untreated and melatonin‐treated cells.

**TABLE 2:** Concentration (EVs/mL) and size (nm) of EVs isolated from breast cancer cell lines (MCF‐7, MDA‐MB‐231, MDA‐MB‐453, and HCC70).

EV source	Concentration (EVs/mL)	Size (nm)
MCF‐7 (untreated cells)	1.74 × 10^10^	133.4
MCF‐7 (melatonin‐treated cells)	2.09 × 10^9^	142.1
MDA‐MB‐231 (untreated cells)	2.33 × 10^9^	152.6
MDA‐MB‐231 (melatonin‐treated cells)	8.21 × 10^8^	142.2
MDA‐MB‐453 (untreated cells)	1.72 × 10^9^	117.0
MDA‐MB‐453 (melatonin‐treated cells)	2.09 × 10^9^	154.1
HCC70 (untreated cells)	2.33 × 10^9^	128.1
HCC70 (melatonin‐treated cells)	8.21 × 10^8^	154.1

Melatonin exposure resulted in differential effects on EV concentration depending on the cell line. EV concentration increased in MDA‐MB‐453 cells, whereas a reduction was observed in MCF‐7, MDA‐MB‐231, and HCC70 cells (Table 2).

**FIGURE 2:**
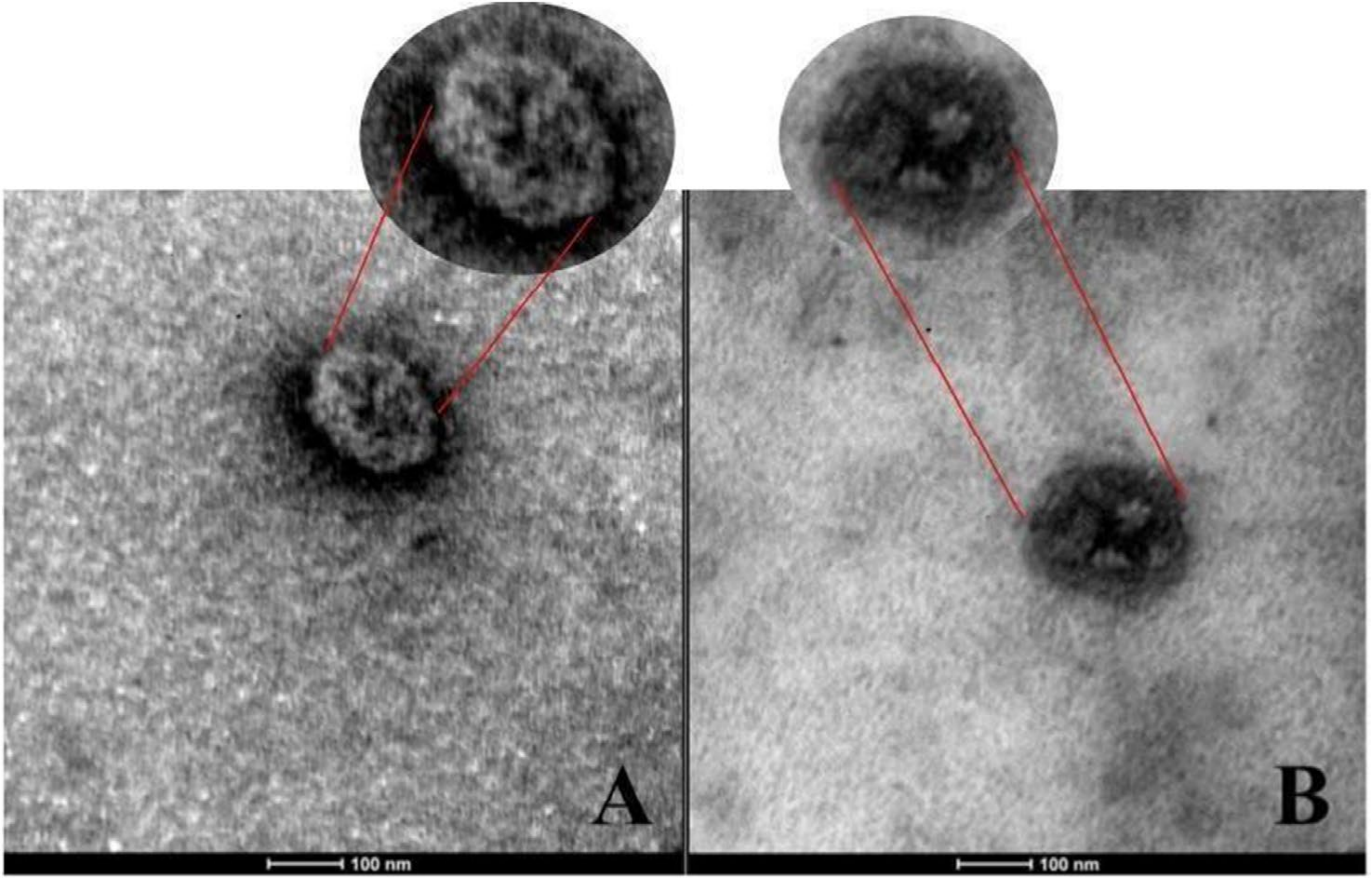
Characterization of extracellular vesicles (EVs) derived from breast cancer cell lines by transmission electron microscopy (TEM). (A) EVs from untreated breast cancer cells; (B) EVs from melatonin‐treated breast cancer cells. Scale bar: 100 nm.

TEM imaging confirmed the characteristic cup‐shaped morphology of EVs, with an average diameter of approximately 100 nm (Figure 2). EVs from untreated cells exhibited lower electron density, while EVs from melatonin‐treated cells showed increased electron density. Overall, EV size and morphology were preserved, while concentration varied in a cell line–dependent manner.

### 3.2 Effect of Extracellular Vesicles Derived From Melatonin‐Treated Cells on Cell Viability in MCF10A Cells

EVs were standardized by particle number prior to treatment, ensuring equivalent vesicle quantities across experimental conditions. Therefore, observed differences are unlikely to reflect EV abundance, but rather EV‐mediated biological effects.

**FIGURE 3:**
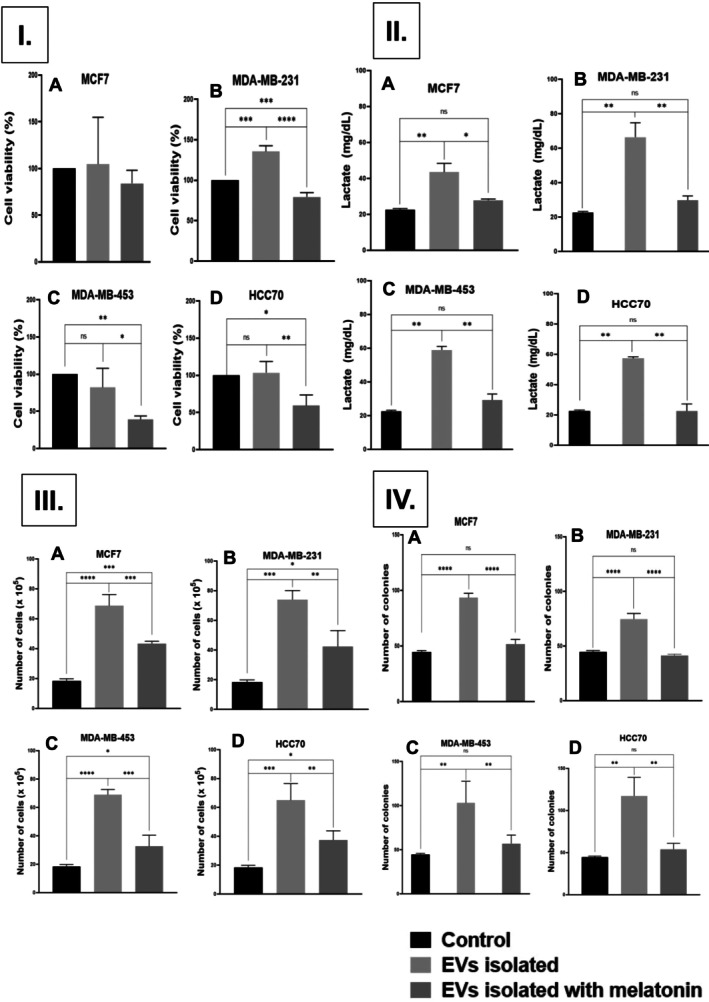
Effects of extracellular vesicles derived from untreated or melatonin‐treated breast cancer cells on viability, migration, colony formation, and lactate production in MCF10A cells. (I) Cell viability of MCF10A cells following treatment with extracellular vesicles (EVs) derived from different breast cancer cell lines cultured under untreated or melatonin‐treated conditions: (A) MCF‐7: (i) Control, (ii) EVs from untreated cells, (iii) EVs from melatonin‐treated cells; (B) MDA‐MB‐231: (i) Control, (ii) EVs from untreated cells, (iii) EVs from melatonin‐treated cells; (C) MDA‐MB‐453: (i) Control, (ii) EVs from untreated cells, (iii) EVs from melatonin‐treated cells; (D) HCC70: (i) Control, (ii) EVs from untreated cells, (iii) EVs from melatonin‐treated cells. Statistical significance is indicated as follows: (*) *p* < 0.05; (**) *p* < 0.01; (***) *p* < 0.001; (****) *p* < 0.0001 compared to the control group. Graphs were generated using GraphPad Prism 9. The *Y*‐axis represents the percentage of viable cells and the *X*‐axis represents experimental conditions. (II) Lactate production in MCF10A cells following treatment with EVs derived from different breast cancer cell lines cultured under untreated or melatonin‐treated conditions: (A) MCF‐7: (i) Control, (ii) EVs from untreated cells, (iii) EVs from melatonin‐treated cells; (B) MDA‐MB‐231: (i) Control, (ii) EVs from untreated cells, (iii) EVs from melatonin‐treated cells; (C) MDA‐MB‐453: (i) Control, (ii) EVs from untreated cells, (iii) EVs from melatonin‐treated cells; (D) HCC70: (i) Control, (ii) EVs from untreated cells, (iii) EVs from melatonin‐treated cells. Statistical significance is indicated as follows: (*) *p* < 0.05; (**) *p* < 0.01; (***) *p* < 0.001; (****) *p* < 0.0001 compared to the control group. Graphs were generated using GraphPad Prism 9. The *Y*‐axis represents lactate levels (mg/dL) and the *X*‐axis represents experimental conditions. (IIII). Cell migration of MCF10A cells following treatment with EVs derived from different breast cancer cell lines cultured under untreated or melatonin‐treated conditions: (A) MCF‐7: (i) Control, (ii) EVs from untreated cells, (iii) EVs from melatonin‐treated cells; (B) MDA‐MB‐231: (i) Control, (ii) EVs from untreated cells, (iii) EVs from melatonin‐treated cells; (C) MDA‐MB‐453: (i) Control, (ii) EVs from untreated cells, (iii) EVs from melatonin‐treated cells; (D) HCC70: (i) Control, (ii) EVs from untreated cells, (iii) EVs from melatonin‐treated cells. Statistical significance is indicated as follows: (*) *p* < 0.05; (**) *p* < 0.01; (***) *p* < 0.001; (****) *p* < 0.0001 compared to the control group. Graphs were generated using GraphPad Prism 9. The *Y*‐axis represents the number of migrated cells and the *X*‐axis represents experimental conditions. (IV) Colony formation of MCF10A cells following treatment with EVs derived from different breast cancer cell lines cultured under untreated or melatonin‐treated conditions: (A) MCF‐7: (i) Control, (ii) EVs from untreated cells, (iii) EVs from melatonin‐treated cells; (B) MDA‐MB‐231: (i) Control, (ii) EVs from untreated cells, (iii) EVs from melatonin‐treated cells; (C) MDA‐MB‐453: (i) Control, (ii) EVs from untreated cells, (iii) EVs from melatonin‐treated cells; (D) HCC70: (i) Control, (ii) EVs from untreated cells, (iii) EVs from melatonin‐treated cells. Statistical significance is indicated as follows: (*) *p* < 0.05; (**) *p* < 0.01; (***) *p* < 0.001; (****) *p* < 0.0001 compared to the control group. Graphs were generated using GraphPad Prism 9. The *Y*‐axis represents the number of colonies and the *X*‐axis represents experimental conditions. Data are presented as mean ± SEM (*n* = 3 independent experiments).

Cell viability was assessed using the MTT assay. EVs derived from melatonin‐treated cells were associated with reduced viability compared to EVs from untreated cells and control conditions. In contrast, EVs from untreated MDA‐MB‐231 and HCC70 cells were associated with increased viability, with statistical significance observed for MDA‐MB‐231. EVs from MDA‐MB‐453 cells were associated with reduced viability (Figure 3I).

### 3.3 Effect of Extracellular Vesicles Derived From Melatonin‐Treated Breast Cancer Cells on Migration of MCF10A Cells

Cell migration was evaluated in MCF10A cells following treatment with EVs derived from malignant breast cancer cell lines, obtained from untreated or melatonin‐treated cells.

EVs derived from melatonin‐treated cells were associated with reduced cell migration compared to EVs derived from untreated cells. Statistical analysis after 24 h showed a significant decrease in the number of migrated cells in MCF10A cultures treated with EVs from melatonin‐treated cells (Figure 3II).

### 3.4 Effect of Extracellular Vesicles Derived From Melatonin‐Treated Cells on Colony Formation in MCF10A Cells

MCF10A cells co‐cultured with EVs derived from breast cancer cell lines (MCF‐7, MDA‐MB‐231, MDA‐MB‐453, and HCC70), obtained from untreated or melatonin‐treated cells, were evaluated for colony formation.

EVs derived from melatonin‐treated cells were associated with a reduction in colony formation compared to EVs from untreated cells. When analysed by cell line, EVs derived from MCF‐7, MDA‐MB‐453, and HCC70 cells showed a slight increase in colony formation following melatonin treatment, whereas EVs from MDA‐MB‐231 cells were associated with decreased colony formation. However, these differences did not reach statistical significance (Figure 3III).

### 3.5 Effect of Extracellular Vesicles Derived From Melatonin‐Treated Cells on Lactate Production in MCF10A Cells

MCF10A cells co‐cultured with EVs derived from malignant breast cancer cell lines, obtained from untreated or melatonin‐treated cells, were evaluated for lactate production. EVs derived from melatonin‐treated cells were associated with decreased lactate levels in MCF10A cells, with values comparable to those observed in control cells. In contrast, EVs derived from untreated malignant cells were associated with increased lactate production (Figure 3IV).

### 3.6 Effect of Melatonin‐Treated Extracellular Vesicles on Apoptosis in Benign Cells

**FIGURE 4:**
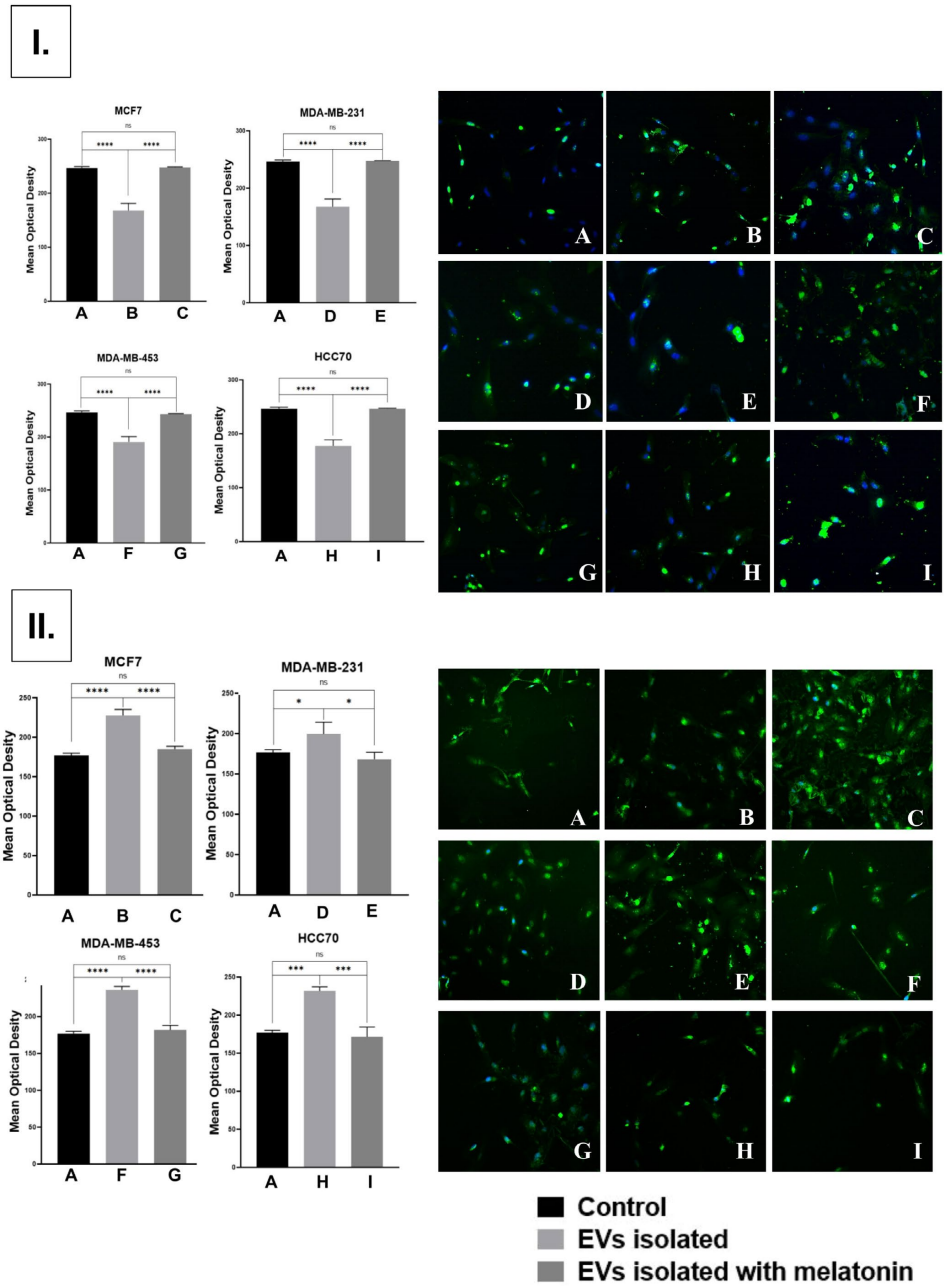
Effects of extracellular vesicles derived from untreated or melatonin‐treated breast cancer cells on apoptosis and proliferation markers in MCF10A cells. (I) Caspase‐3 activity in MCF10A cells following treatment with EVs derived from breast cancer cell lines cultured under untreated or melatonin‐treated conditions. For each cell line: (i) Control, (ii) EVs from untreated cells, (iii) EVs from melatonin‐treated cells. Statistical significance is indicated as follows: (*) *p* < 0.05; (**) *p* < 0.01; (***) *p* < 0.001; (****) *p* < 0.0001 compared to the control group. The *Y*‐axis represents mean optical density. Immunofluorescence images: (A) Control; (B–I) corresponding to EV‐treated conditions from each breast cancer cell line. Green: cleaved caspase‐3; Blue: DAPI. (II) Ki‐67 expression in MCF10A cells following treatment with EVs derived from breast cancer cell lines cultured under untreated or melatonin‐treated conditions. The *Y*‐axis represents mean optical density. Immunofluorescence images: (A) Control; (B–I) corresponding EV treatments. Green: Ki‐67; Blue: DAPI. Data are presented as mean ± SEM (*n* = 3 independent experiments).

Apoptosis was evaluated in MCF10A cells following treatment with EVs derived from malignant breast cancer cell lines, obtained from untreated or melatonin‐treated cells. MCF10A cells treated with EVs derived from untreated malignant cells exhibited reduced apoptotic activity, as indicated by decreased cleaved caspase‐3 expression. In contrast, cells treated with EVs derived from melatonin‐treated cells showed increased cleaved caspase‐3 levels, with values comparable to those observed in control cells (Figure 4I).

### 3.7 Effect of EVs Derived From Melatonin‐Treated Cells on Ki‐67 Expression in Benign Breast Cells

Ki‐67 expression was evaluated as a marker of cell proliferation in MCF10A cells following treatment with EVs derived from malignant breast cancer cell lines, obtained from untreated or melatonin‐treated cells.

Immunofluorescence analysis showed that MCF10A cells treated with EVs derived from melatonin‐treated cells exhibited significantly reduced Ki‐67 expression compared to cells treated with EVs from untreated cells. This reduction was consistently observed across all EVs derived from the different breast cancer cell lines (Figure 4II).

## 4 Discussion

This study demonstrates that extracellular vesicles (EVs) derived from malignant breast cancer cells are capable of modulating the phenotype of benign mammary cells, promoting functional alterations associated with increased proliferation, migration, and metabolic activity. Importantly, EVs derived from melatonin‐treated tumour cells were associated with attenuation of these effects, indicating that exposure of donor cells to melatonin alters EV‐mediated intercellular communication. These effects should be interpreted as being mediated by extracellular vesicles derived from melatonin‐treated donor cells, rather than a direct effect of melatonin on recipient cells.

The characterization of EVs confirmed size distribution and morphology consistent with established guidelines, with no major differences between EVs derived from untreated and melatonin‐treated cells. However, melatonin exposure resulted in differential effects on EV concentration depending on the cell line. EV production increased in MDA‐MB‐453 cells, whereas a reduction was observed in MCF‐7, MDA‐MB‐231, and HCC70 cells. Although these findings may appear contradictory at first, they indicate that the effect of melatonin on EV biogenesis [20, 21] is not uniform and is likely dependent on the intrinsic molecular and metabolic characteristics of each breast cancer subtype, including differences in receptor expression, signalling pathways, and metabolic state.

All experimental conditions were standardized based on EV particle number prior to treatment. Therefore, the functional differences observed in recipient MCF10A cells are unlikely to be driven by variations in EV quantity, but rather reflect qualitative differences in EV cargo and biological activity [22, 23, 24]. In this context, the divergent effects of melatonin on EV production reinforce the notion that its primary impact in this study is related to modulation of EV composition and function, rather than simply altering vesicle abundance.

The functional assays indicate that EVs derived from malignant cells can induce phenotypic reprogramming in benign MCF10A cells. Increased viability, migration, colony formation, and lactate production observed in response to EVs from untreated tumour cells are consistent with previous reports demonstrating that tumour‐derived EVs promote pro‐tumoural traits in recipient cells [4, 6, 25, 26]. In particular, the increase in lactate production supports the notion that EVs contribute to metabolic reprogramming toward a glycolytic phenotype [23, 25, 27], a hallmark of cancer‐associated metabolic adaptation.

In contrast, EVs derived from melatonin‐treated cells were associated with reduced proliferation, migration, and metabolic activity in MCF10A cells. These findings suggest that melatonin exposure modifies the biological activity of EVs [9, 28], reducing their capacity to induce aggressive phenotypes.

The mechanisms underlying these observations were not directly investigated; however, it is plausible that melatonin exposure alters the molecular cargo of EVs, including proteins, lipids, and regulatory RNAs. Previous studies have shown that melatonin can modulate EV composition [29, 30] and influence signalling pathways in recipient cells [9, 15, 28], supporting the hypothesis that changes in EV cargo [22, 23, 24] contribute to the functional effects observed in this study.

Consistent with this interpretation, EVs derived from melatonin‐treated cells were associated with reduced lactate production and increased caspase‐3 activity in MCF10A cells, suggesting a shift toward a less glycolytic and more apoptosis‐prone phenotype [9, 12]. Additionally, decreased Ki‐67 expression indicates reduced proliferative activity, further supporting the notion that melatonin‐modulated EVs attenuate pro‐tumoural signalling.

Despite these findings, several limitations should be considered. Although EVs were standardized by particle number prior to treatment, EV uptake by recipient cells was not directly quantified. Therefore, differences in cellular responses may partially reflect variations in EV internalization efficiency between experimental conditions. In addition, the molecular cargo of EVs was not characterized, preventing identification of the specific mediators responsible for the observed effects.

In the migration assay, serum was present in the lower chamber to support cell viability and promote migration; however, the presence of serum in the experimental setup may have influenced the establishment of an optimal chemotactic gradient. Although this condition may limit the precise assessment of directed chemotactic migration, relative differences between experimental groups remain informative of directed migration. It was maintained to ensure adequate cell survival and reproducibility across experimental conditions. Therefore, the observed differences in migration should be interpreted with caution, as they may reflect both EV‐mediated effects and potential influences of serum conditions. Although a serum‐free condition throughout the assay was not included, all experimental groups were subjected to identical conditions, allowing for relative comparisons between treatments.

From a mechanistic perspective, considering the well‐established antioxidant properties of melatonina [9, 12], it is plausible that exposure of donor tumour cells to melatonin may influence the redox state of these cells and, consequently, alter the molecular cargo of EVs [22, 24, 30]. Such changes may affect redox‐related signalling pathways in recipient cells, contributing to the observed modulation of proliferation, metabolism, and apoptosis. In this context, future studies should investigate parameters such as reactive oxygen species (ROS) production and EV‐associated redox‐regulating molecules to further elucidate these mechanisms.

Collectively, these findings support the concept that tumour‐derived EVs play a significant role [4, 6, 21, 25, 26] in modulating the behaviour of surrounding cells and that exposure of tumour cells to melatonin can alter EV‐mediated signalling, reducing their pro‐tumoural impact. This study expands the current understanding of EV‐dependent communication in breast cancer and highlights the potential of targeting EV‐associated pathways [21, 31] as a strategy to modulate tumour–microenvironment interactions.

## 5 Conclusions

The interaction between melatonin and extracellular vesicles (EVs) represents a promising area of investigation in cancer biology. The present study provides evidence that EVs derived from malignant breast cancer cells can induce functional and phenotypic alterations in benign mammary cells, including increased proliferation, migration, and metabolic changes.

EVs derived from melatonin‐treated malignant cells were associated with attenuation of these effects, indicating that exposure of donor tumour cells to melatonin may modulate EV‐mediated intercellular communication and influence their impact on recipient cells.

Although the molecular mechanisms underlying these observations were not directly investigated, the findings support the hypothesis that melatonin exposure may alter EV‐associated signalling. However, further studies are required to characterize the molecular cargo of EVs and to elucidate the mechanisms involved.

Overall, this study contributes to the understanding of EV‐mediated communication in breast cancer and highlights the potential relevance of targeting EV‐associated pathways within the tumour microenvironment.

## Author Contributions

**Barbara Maria Frigieri:** conceptualization, investigation, writing – original draft, methodology, project administration, data curation. **Adriana Alonso Novais:** conceptualization, writing – original draft, validation, visualization, writing – review and editing. **Caroline Patini de Rezende:** investigation, validation, methodology, data curation, supervision, writing – review and editing. **Mari Cleide Sogayar:** investigation, writing – review and editing, validation, project administration, supervision. **Guilherme Silva Bruno Barbosa:** investigation, methodology. **Debora Aparecida Pires de Campos Zuccari:** conceptualization, funding acquisition, writing – original draft, writing – review and editing, visualization, validation, project administration, resources, supervision. **Vinicius Augusto Simão:** investigation, visualization, validation, methodology, data curation. **Fausto Almeida:** investigation, writing – review and editing, methodology, formal analysis, supervision. **Russel J. Reiter:** investigation, writing – review and editing, data curation, supervision. **Luiz Gustavo de Almeida Chuffa:** investigation, writing – review and editing, writing – original draft, formal analysis, supervision, data curation.

## Funding

This work was financially supported by the Fundação de Amparo a Pesquisa do Esdado de São Paulo (FAPESP), grant number 2020/12970‐8 to DAPCZ, FAPESP Thematic grant number 2016/05311‐2 to MCS, by the Coordenação de Aperfeiçoamento de Pessoal de Nível Superior (CAPES), Finance Code 88887.707044/2022‐00 and the Conselho Nacional de Desevolvimento Cientfico e Tecnologico (CNPQ) to the process numbers 306117/2023‐1 to LGAC and 307288/2022‐6 to DAPCZ.

## Ethics Statement

This study was conducted using established human breast cell lines and, therefore, was exempt from review by the local ethics committee. The MCF7 and HCC70 cell lines are part of our laboratory's existing cell bank. The MDA‐MB‐231 and MDA‐MB‐453 cell lines were kindly provided by Dr. Guilherme Henrique Tamarindo, a researcher at the Brazilian Biosciences National Laboratory (LNBio). The MCF10A cell line was generously provided by Professor Dr. Thomas Ong, Associate Professor at the University of São Paulo (USP), and was cultivated at the NUCEL Laboratory under the supervision of Professor Dr. Mari Cleide Sogayar, also at USP.

## Conflicts of Interest

The authors declare no conflicts of interest.

## Data Availability

The data used to support the findings of this study are available from the corresponding author upon request.
